# Reverse Shoulder Arthroplasty Humeral Lateralisation: A Systematic Review

**DOI:** 10.7759/cureus.19845

**Published:** 2021-11-23

**Authors:** Govind Dhillon, Madeline Warren, Angelos Assiotis, Adam Rumian, Harpal S Uppal

**Affiliations:** 1 Trauma and Orthopaedics, Lister Hospital, Stevenage, GBR; 2 Trauma and Orthopaedics, Bristol Royal Infirmary, Bristol, GBR; 3 Trauma and Orthopaedics, Lister Hospital, East and North Hertfordshire Trust, Stevenage, GBR

**Keywords:** reverse shoulder arthroplasty, centre of rotation, ascend flex, 145, 135, 155, lateralisation, inlay, onlay

## Abstract

Different studies on reverse shoulder arthroplasty (RSA) have proposed changes to the humeral design to lateralise the humeral centre of rotation (COR), with humeral inclination to 135 or 145 from 155 degrees or to switch to onlay humeral trays from inlay design; with both having also been used in combination. There have been many studies and systematic reviews to show the difference in outcomes and complications to the variations in glenoid design but to date, there have been no systematic studies to compare different humeral inclinations for RSA implants.

Searches using keywords were used in common medical search engines in a systematic fashion. The article was reviewed for the class of evidence and bias, summarised and compared in meta-analysis. Inclusion criteria included studies on adults with RSA that compared lateralised humeral implants to medialised.

The search produced 349 articles; of these, we identified nine studies that met the inclusion criteria. Our review identified a total of 562 patients who had been included in studies directly comparing lateralised humerus to a more medial design. Meta-analysis showed a significantly reduced risk of scapular notching in lateralised humerus compared to the standard medialised component. The external rotation range of motion in the lateralised group was statistically significant.

The improvement in scapular notching and gain in the range of motion without any apparent downside in the form of reduced patient-reported outcome measures or complications suggest a lateralised humeral component is superior to the more medialised design in RSA. A large RCT with a longer-term follow-up is needed to confirm whether there is clinically significant benefit from the lateralisation of the humerus.

## Introduction and background

The glenohumeral joint is prone to many disabling conditions, including rotator cuff tears, rotator cuff tear arthropathy, shoulder osteoarthritis, glenoid bone loss, and proximal humerus fracture [1-3]. Reverse shoulder arthroplasty (RSA) is a frequently used treatment for these conditions and generally gives good clinical outcomes where conservative measures may fail. The number of reverse shoulder arthroplasty procedures performed in the UK has been increasing steadily since National Joint Registry (NJR) first began recording in 2012 [4]. A total of 4,512 primary reverse shoulder replacements were performed in 2019, a year-on-year increase of 13.6% from 2018, only dropping in 2020 due to coronavirus disease 2019 (COVID-19) [4].

While RSA has been in use since the 1970s, many biomechanical variations of designs have taken place since their introduction. Early designs aimed to mimic the anatomical centre of rotation (COR) [5]. The design of RSA was popularised by the Grammont design that medialises both the humerus and the glenoid [6]. The medialised designs have many theoretical and clinical advantages over earlier generation designs. Advantages include reduced mechanical torque at the glenoid joint surface, which reduces glenoid loosening and improves deltoid tension and joint stability by increased humeral length, whilst the medialised glenoid COR increases the abductor lever arm, which improves deltoid efficiency [6]. The massive increase in the use of the RSA over the last three decades has been driven by these innovations, such that the centre of rotation of glenoid components in most modern RSA is medialised compared to the anatomical COR.

The Grammont medialised glenoid design shows good long-term outcomes and function, however, up to half of all patients develop scapular notching with no improvement or even loss in active external rotation [7-8]. There are concerns that these complications are caused by excessive medialisation of the RSA by creating bony impingement, impairing anterior and posterior deltoid function and defunctioning the remaining anterior and posterior rotator cuff. These concerns have led to the development of differing designs, which vary the degree of medialisation [9]. Most of these efforts have been directed at lateralising the glenosphere as compared to a Grammont style implant. These techniques may involve the build-up of the glenosphere with a metal augment; the use of a glenosphere comprises more than a 180-degree arc of a sphere. Also with the use of humeral bone graft underneath the base plate, called a bony increased offset (BIO)-RSA, as popularised by Boileau. BIO-RSA increases the lateralisation of the glenosphere by increased lateral and associated inferior offset. The prospective study by Boileau compares the lateralised glenoid centre of rotation with standard designs [10]. The study showed improved outcomes by increasing scapular neck length and increasing external rotator/internal rotator tension for better stability and rotation. However, decreasing impingement is at the risk of increased loosening [10].

Different studies have proposed changes to the humeral design to lateralise the humeral COR by altering the humeral inclination to 135 or 145 from 155 degrees or to switch to onlay humeral trays from inlay design; with both having also been used in combination [11-13]. Onlay designs have the advantage in comprehensive usage and less bone loss [11-13]. In 2008, Gutierrez et al. created a three-dimensional (3D) model of an RSA and was able to demonstrate that the most important implant factor for reduced notching was lateralised humeral COR followed by inferior positioning [14]. Cadaveric studies have shown increased dislocation force required of a 135-degree neck-shaft angle (NSA) in the externally rotated position but favourable results towards 155 degrees in the more prone internally rotated position [15]. This improvement was later supported by Ascione et al. with 485 implants in live patients showing significantly improved active motion and Constant Murley scores, although 21 cases (4.3%) of scapular spine fracture were also reported [16]. The Constant score and American shoulder and elbow surgeons standard shoulder assessment form (ASES) are the two most commonly used scores in studies, with constant shown to reliably detect improvement after intervention [17-18].

There have been many studies and systematic reviews to show the difference in outcomes and complications to the variations in glenoid design but to date, there have been no systematic studies to compare different humeral inclinations for RSA implants. The purpose of this study is to systematically review the literature on RSA to determine if modern lateralised humeral stems confer an advantage over the traditional Grammont style humeral stem. 

## Review

Methods

Search Strategy

The protocol was prepared a priori and registered with Prospero (registration number: CRD42021259950) in June 2021 before initial searches had taken place. A systematic review and meta-analysis of patient outcomes were conducted in accordance with the Preferred Reporting Items for Systematic Reviews and Meta-Analyses (PRISMA) guidelines [19].

We systematically searched the MEDLINE/PubMed, EMBASE-Ovid, and Cochrane databases using the above-mentioned keywords, MeSH terms, and keywords and synonyms, as well as combinations of these terms. Specifically, we wished to identify papers reporting outcomes for patients undergoing reverse shoulder arthroplasty directly comparing techniques lateralizing the humeral component to non-lateralized. The search was restricted to English language papers published within the last 10 years.

Inclusion Criteria

Studies that were deemed eligible for inclusion satisfied the following criteria: 1) Humans (>16 years), 2) RSA operation, 3) Minimum one-year follow-up, 4) Lateralized humeral implant vs Medialised/Standard/Grammont, 5) Patient outcome reported.

Exclusion Criteria

Studies were excluded if they included any of the following: 1) Cadaveric or animal study, 2) Glenoid lateralized implant studies, 3) Non-comparative case series or studies with incorrect comparator, 4) Classification studies, 5) Morphology studies, 6) Simulation studies, 7) Review articles.

Screening

Two review authors (MW and GD) independently screened titles and abstracts using the Covidence software [20]. Where eligibility was unclear, the full text was retrieved and assessed. Disagreements were resolved by the third supervising author (HU).

Assessment of Bias

As the majority of included studies were non-randomised, two reviewers (MW, GD) independently assessed for risk of bias using the Newcastle-Ottawa quality assessment tool once irrelevant papers were excluded [21].

Data Analysis

A meta-analysis was performed using a fixed-effects model unless measures of heterogeneity were high with Review Manager 5.4.1 (The Cochrane Collaboration, 2020) [22]. Outcome measures included postoperative Constant scores, ASES, rate of scapular notching and post-operative external rotation for the lateralized humerus versus standard care.

Results

**Figure 1:**
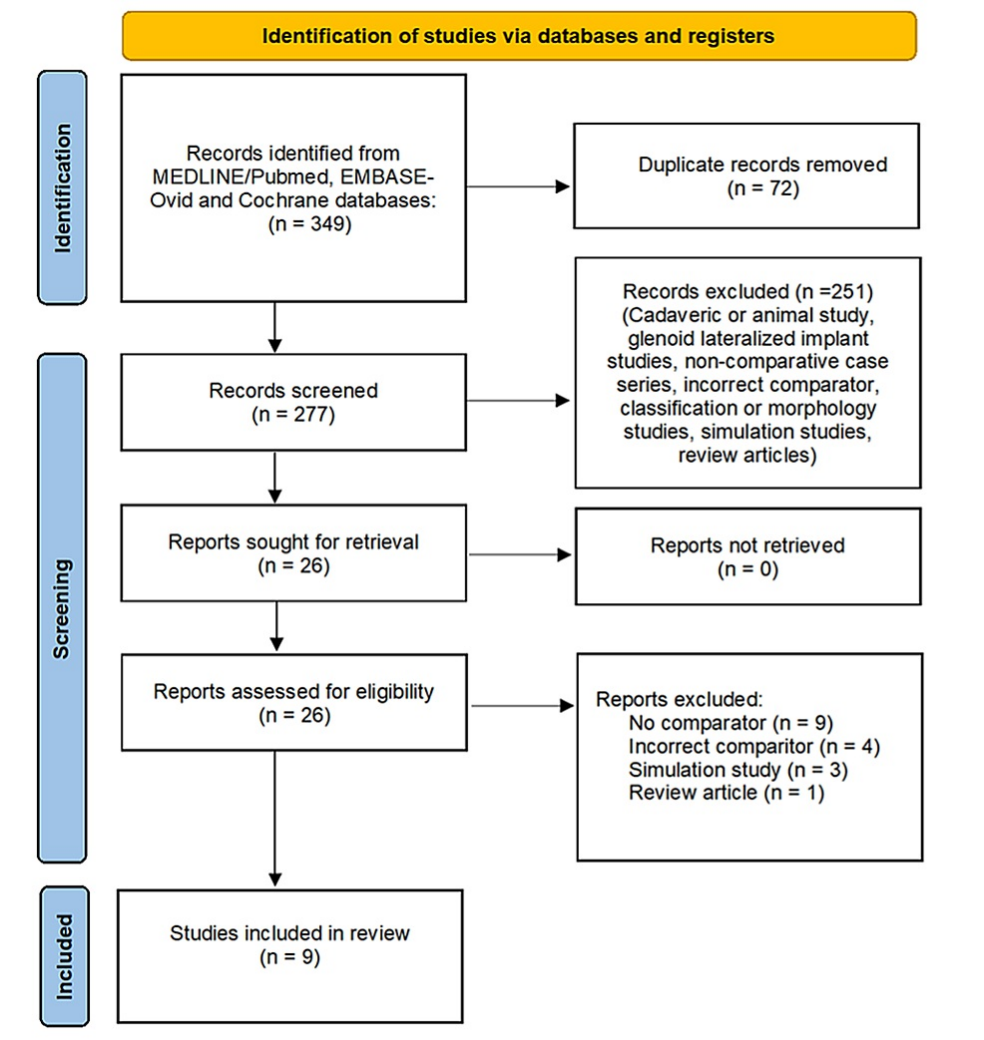
PRISMA flow chart showing search strategy and outcome Source: [19] PRISMA: Preferred Reporting Items for Systematic Reviews and Meta-Analyses

A total of 349 references were identified, which were reduced to 17 papers for full-text review after removal of duplicates and application of exclusion criteria (Figure 1) [19]. Of these, nine papers met the full inclusion criteria (Table 1) [23-31].

**Table 1:** Summary of studies included for meta-analysis Studies included in table [23-31] no of pt = number of patients, BIO-RSA = bony increased offset-reversed shoulder arthroplasty, NSA = Neck shaft angle, Ascend flex = Aequalis Ascend™ Flex, ASES = American Shoulder and Elbow Surgeons Shoulder Score, L = Lateralised, M = Medialised Quality assessment was done via the Newcastle-Ottawa score.

Article	Year	Level of evidence	Quality Assessment [21]	Patients (Male/Female)		Implant		Follow up (Months)	Constant score change		ASES score change		External rotation change (degrees)		Scapular notching (no of pt and %)		
				L	M	L	M		L	M	L	M	L	M	L	M	
Beltrame [23]	2019	3A	Good (4, 1, 3)	21 (6/15)	21 (6/15)	Ascend flex	Grammont	12	+32	+31			+21	+18	0/21 (0%)	3/21 (15%)	
Boutsiadis (A) [24]	2018	3A	Good (4, 2, 2)	10	13	Ascend flex +Standard base	Grammont + Standard base	12	+46	+39	+67	+75	+32	+0	-	-	
Boutsiadis (B) [24]	2018	3A	Good (4, 2, 2)	12	11	Ascend flex + Bio RSA base	Grammont + Bio RSA base	12	+38	+46	+62	+65	+19	+19	-	-	
Gobezie [25]	2019	2B	Good (4, 1, 2)	37 (14/23)	31 (9/22)	135 NSA	155 NSA	24	-	-	37	41	+1	+1	8/37 (21%)	18/31 (58%)	
Merolla [26]	2018	3A	Good (3, 1, 3)	38 (13/25)	36 (10/26)	Ascend Flex	Grammont	24	+44.2	+51.8	-	-	+32	+15	2/38 (5%)	14/36 (39%)	
Nelson [27]	2018	3A	Good (4, 1, 3)	49 (13/36)	48 (21/27)	135 NSA	155 NSA	12	-	-	+51	+41.6	+13.1	+6	6/46 (12%)	17/48 (35%)	
Zitovsky [31]	2020	3A	Good (4, 2, 3)	68 (20/48)	39 (18/21)	135 NSA	155 NSA	24			+55.1	+45.8	+24.6	+12.76	10/68 (14.7%)	16 /39 (40%)	
Paper	Year	Level of evidence		Patients (Male/Female)		Implant		Follow up (Months)	Constant score post-operative		ASES score post-operative		External rotation post-operative (Degrees)		Scapular notching (no of pt and %)		
				L	M	L	M		L	M	L	M	L	M	L	M	
Polisetty [28]	2020	3A	Good (4, 1, 2)	46 (22/24)	46 (18/28)	Onlay stryker	Inlay Altivate	24	-	-	78	80.1	49.1	38	4/46 (9%)	4/46 (9%)	
Schoch [29]	2020	3A	Good (3, 1, 2)	125 (54/71)	17 (6/11)	Equinoxe (Exactech)	Grammont a) Aqualis (13pt) b) Delta III (4pt)	36	71.7	69.9	75.5	76.4	26	24	28/125 (22%)	14/17 (82%)	
Verdano [30]	2018	3A	Poor (4, 0, 2)	16	16	Equinoxe	Grammont	14.3	61	64	-	-	19, 41	12.7, 35	0/16 (0%)	3/16 (19%)	

All articles included studies that were either prospective cohorts or retrospective reviews, with the exception of one randomized controlled trial by Gobezie et al. [25].

Quality assessment using the Newcastle-Ottawa scale was good for all papers with the exception of Verdano et al. 2018, which had a follow-up period of only six months and excluded patients who were not compliant with physiotherapy and did not describe demographics [30]. In a sensitivity test, the removal of the Verdano et al. study paper did not significantly impact the outcome of results [30].

Scapular Notching

**Figure 2:**
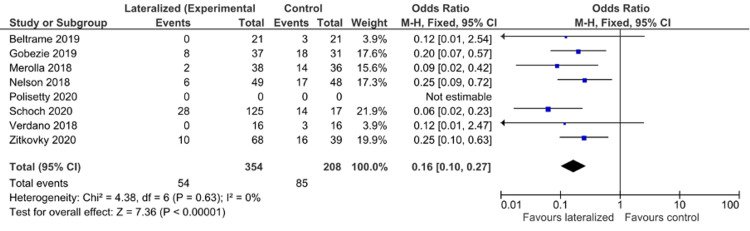
Scapular notching forest plot [23,25-26,28-31]

The presence of scapular notching was reported by seven of the authors, including a total of 562 patients between them. The combined result shows that lateralization of the humeral component gives a significant reduction in the presence of scapular notching at follow-up with an odds ratio of 0.17 (95% CI 0.10, 0.28) (Figure 2).

Constant Scores

**Figure 3:**
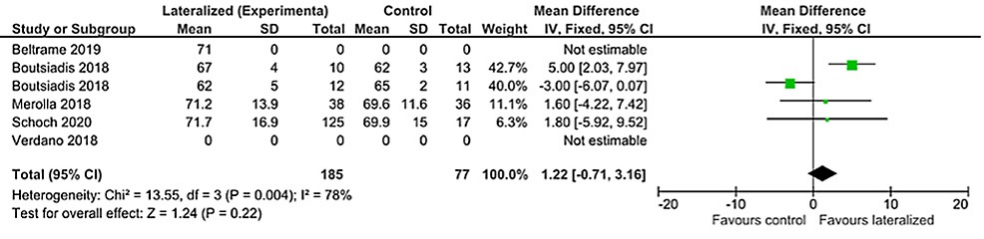
Constant score forest plot [23-24,26,29-30]

Post-operative constant scores were reported adequately in four of the included papers to directly compare outcomes for lateralized humeral components versus standard care, including a total of 262 patients. Results reported by Boutsiadis et al., included four groups, two with lateralized glenoids and two without so these are reported as two separate comparisons [24]. Measures for heterogeneity were high so a random-effects model was used to compare outcomes. This showed no significant difference between lateralized and non-lateralized constant scores for these papers (Figure 3).

ASES

**Figure 4:**
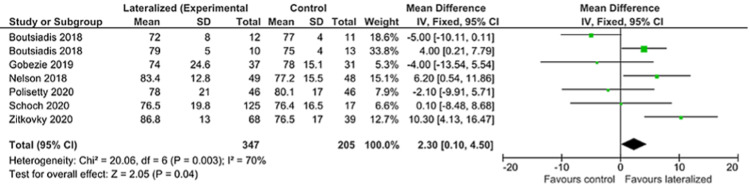
American Shoulder and Elbow Surgeons shoulder score (ASES) forest plot [24-25,27-29,31]

Six authors reported ASES scores for their patients, including a total of 552 patients. There was also no significant difference between ASES scores between lateralized and non-lateralized groups (Figure 4).

External Rotation

**Figure 5:**
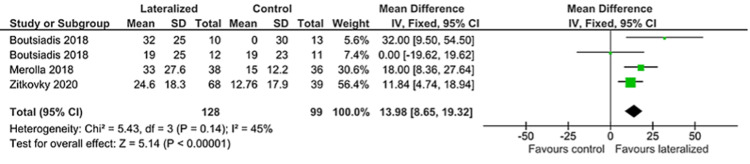
External rotation forest plot (1/2) [24,26,31]

**Figure 6:**
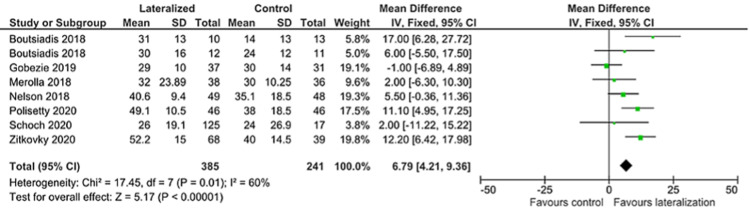
External rotation forest plot 2/2 [24-29,31]

Seven authors published values for post-operative external rotation, of these three also reported gain in external rotation. Aggregated, these papers show a greater gain in external rotation for lateralized humeral components of 14 degrees (95% CI 8.65, 19.32). The post-operative absolute value in measured external rotation remained higher for the lateralized components when aggregated for all seven studies, but only 7 degrees difference between the two groups (95% CI 4.21, 9.36) (Figures 5-6).

Complications

Scapular notching was the most frequent complication presented in all but one paper, Boutsiadis et al. (2018), which did not report post-operative complications [24]. Heterotrophic ossification was reported in three papers [27,30-31] with a total of 35 cases in the lateralised group and 54 cases in the medialised group [27,30-31]. Poisetty et al. [28] reported bone resorption in both groups; greater tuberosity (GT) and calcar resorption occurred in 34 and 18 patients, respectively, for the lateralised group, with the medialised group having 13 for GT and only one for calcar.

Acromial fractures were the most commonly reported fractures, the largest number reported by Poisetty et al. [28] with six acromial fractures in the lateralised group and four in the medialised group. There was one additional medialised acromial fracture reported by Nelson et al. [27]. The Merolla et al. study reported three fractures in the lateralised group, one acromial fracture and two scapular spine fractures [26]. The Nelson study had one humeral shaft fracture each for the medialised and lateralised groups.

Only three dislocations were reported, two medialised and one lateralised. The lateralised group had four revisions compared to no revisions in the medialised group. Causes for revision were infection, dislocation from the Merolla study [26] and then mechanical clicking and humerus fracture from the Schoch study [29].

Discussion

Our aggregated results show that patients undergoing reverse shoulder arthroplasty with a lateralised humeral component were significantly less likely to develop scapular notching.

The clinical relevance of scapular notching has remained unclear since Levigne et al. first evaluated scapular notching in their 2010 series [32]. It was found that patients with notching experienced worse clinical outcomes than those without; in addition, notching progressed over time [32]. This becomes more relevant as patients undergo RSA at younger ages and are expected to live longer with implants. Jang et al. have since confirmed with a systematic review and meta-analysis that notching is indeed associated with worse patient-reported outcomes and range of movement [33]. Longer time periods of erosion may eventually lead to loosening of components and possibly the need for more complex revision surgery. The lack of any significant difference in other complications is encouraging and supports the continued use of lateralised humeral components.

We found no clinically significant difference in patient-reported outcomes for lateralised humeral components compared to standard medialised components, at least in the years immediately following RSA. The study by Tashijian et al. estimated that a change of ASES score of 17.9 for men and 22.4 for females was needed to achieve the Minimal Clinically Important Difference [14].

Our study encountered a variety of limitations. A variety of measures were reported by different authors, reducing the number of patients for comparison. In addition to this, the length of follow-up was typically around two years; sufficient to evaluate the majority of post-operative complications but possibly not long enough to evaluate the longer-term effects of scapular notching.

Despite this, lateralising the humeral component appears to confer a marginal benefit in the external rotation achieved post-operatively. While this may not yet translate into noticeably better clinical outcomes for the patient, future developments and improvements may extend these gains to the point where this contribution becomes relevant.

Overall, based on the body of evidence available, lateralisation of the humeral component of the RSA appears to confer benefits like deltoid wrapping with better shoulder contour without significantly increasing the risk of complications. To confirm this, future research would benefit from follow up over an extended period and further evaluation of the risk factors and natural progression of scapular notching. The use and reporting of both ASES and constant, range of motion including external rotation in addition to scapular notching and other complications would also improve the aggregation and comparability of future studies [34].

The main drawback of our review is the predominantly observational nature of included papers. Only one RCT has been reported and many of the cohort studies were retrospective, which may explain why we found no clinically significant difference between the groups. For the future, a prospective RCT, adequately powered for superiority on the basis of a validated outcome score such as the ASES score or via kinematic registration of joint position to measure the range of motion, which compares medialised to lateralised humeral stems would be the next step forwards to definitively answering this question.

## Conclusions

Our review identified a total of 562 patients who had been included in studies directly comparing the lateralised humerus to a more medial design. We found a significant reduction in scapular notching in the lateralised humerus compared to the standard medialised component and no apparent difference in other complications between the two groups. While we found no statistically significant difference in patient-reported outcome measures, there was wide variation in authors’ choice of evaluation tools, leading to a small number of studies for each. The identified gains in external rotation were statistically significant. The improvement in scapular notching and gain in the range of motion without any apparent downside in the form of reduced patient report outcome measures or complications suggest a lateralised humeral component is superior to the more medialised design in RSA.

We recommend that authors of future studies report both ASES and constant scores, ROM, including external rotation, as well as scapular notching and other complications. The field would benefit from a large RCT with longer-term follow-up to confirm our findings and identify whether there is clinically significant benefit from the lateralisation of the humerus.
